# The first pineoblastoma case report of a patient with Sotos syndrome harboring NSD1 germline mutation

**DOI:** 10.1186/s12887-024-04636-y

**Published:** 2024-03-08

**Authors:** Xizan Yue, Bo Liu, Tiantian Han, Didi Guo, Ran Ding, Guangyu Wang

**Affiliations:** 1 Department of Neurosurgery, 1 Children’s Hospital Affiliated to Shandong University, 2 Jinan Children’s Hospital, Jinan, China; 2 The State Key Lab of Translational Medicine and Innovative Drug Development, Jiangsu Simcere Diagnostics Co, Ltd, Nanjing, China; 3 The Medical Department, Jiangsu Simcere Diagnostics Co., Ltd, Nanjing Simcere Medical Laboratory Science Co, Ltd, Nanjing, China

**Keywords:** *NSD1*, Sotos syndrome, Pineoblastoma

## Abstract

Germline mutations of *NSD1* are associated with Sotos syndrome, characterized by distinctive facial features, overgrowth, and developmental delay. Approximately 3% of individuals with Sotos syndrome develop tumors. In this study, we describe an infant in pineoblastoma with facial anomalies, learning disability and mild autism at 1 years diagnosed as Sotos syndrome owing to carrying a novel mutation de novo germline *NSD1* likely pathogenic variant. This patient expands both the mutation and phenotype spectrum of the Sotos Syndrome and provides new clinical insights into the potential mechanism of underlying pinealoblastoma pathology.

## Introduction

The nuclear receptor SET domain-containing protein 1 (NSD1) gene, located on 5q35, is one of the genes recently recognized to developmentally regulate the epigenome. It encodes a histone methyltransferase responsible for catalyzing the transfer of methyl groups to lysine residues of histone tails, which is crucial for various aspects of normal embryonic development [1]. Loss-of-function mutations in *NSD1* have been associated with global genome histone methylation changes that disrupt gene expression and have also been reported in head and neck squamous cell carcinoma and leukemia [2, 3].

Germline mutations of *NSD1* are associated with Sotos syndrome (OMIM 117550), characterized by distinctive appearance, overgrowth, and developmental delay [4]. The main features of Sotos syndrome include behavioral problems (most prominently autism spectrum disorders), cardiac abnormalities, cranial MRI/CT abnormalities, advanced skeletal age, joint hyperlaxity with or without flat feet, maternal preeclampsia, renal abnormalities, neonatal complications, scoliosis, and seizures. The global incidence rate is approximately 1:10,000–1:50,000 [5]. Approximately 3% of individuals diagnosed with Sotos syndrome develop various tumors such as sacrococcygeal teratoma, neuroblastoma, presacral ganglioma, astrocytoma, small-cell lung cancer, and acute lymphoblastic leukemia [6–8]. Notably the patient represents the first documented case of pineoblastoma (PB) within the context of Sotos syndrome.

There is limited information on Sotos syndrome in China, with only five cases reported [9]. In this study, we present a case of an infant PB patient exhibiting facial anomalies, learning disability, and mild autism. The diagnosed of Sotos syndrome was confirmed based on the identification of a novel mutation de novo germline *NSD1* likely pathogenic variant.

## Materials and methods

Next generation sequencing (NGS) was performed to analyze postoperative tissue and peripheral blood samples. DNA from both parents was extracted from peripheral blood. Somatic mutations and copy number variations were detected following the standard operating procedure (SOP). The pathogenicity of germline mutations, including SNVs and small INDEL, was categorized based on American College of Medical Genetics and Genomics (ACMG) guidelines.

## Results

**Fig. 1:**
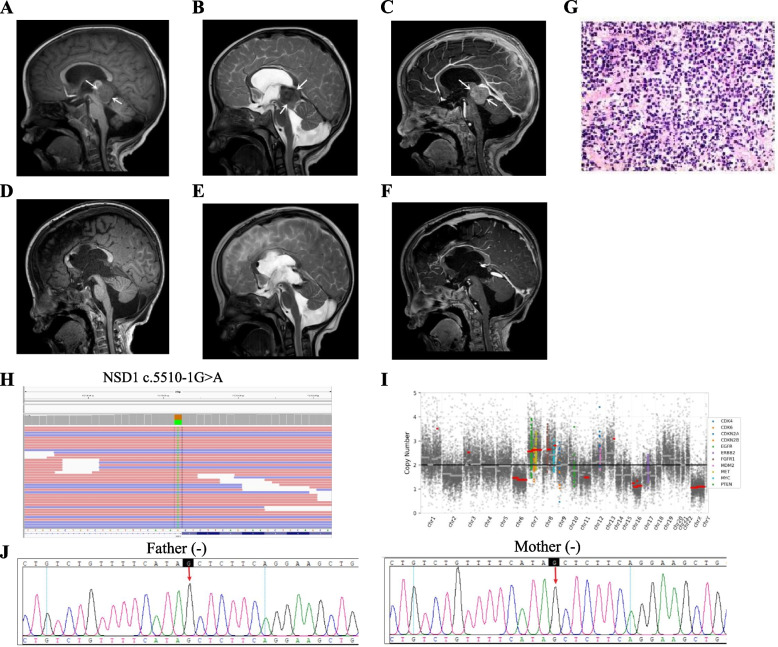
MRI revealed a space-occupying lesion in the preoperative (**A**-**C**) and postoperative (**D**-**F**) pineal region. (**A**), Sagittal T1-weighted image showed that the tumor (arrows) was nearly isointense relative to gray matter. (**B**), Sagittal T2-weighted image showed that the tumor (arrows) was nearly isointense relative to gray matter. (**C**), Sagittal T1-weighted gadolinium-enhanced image showed homogeneous enhancement of the tumor (arrows). After surgery, the tumor disappeared on T1-weighted image (**D**), T2-weighted image (**E**), and T1-weighted gadolinium-enhanced image (**F**). HE staining from pineal regions suggested pineoblastoma (**G**) and NGS revealed comprehensive genomic profiling (**H**, **I**). Sanger sequencing of blood samples from the patient’s parents was carried out for family verification (**J**): Father (-), Mother (-)

The hospital admission of a 1-year-old male infant took place on April 24, 2021, due to a two-day history of nausea and vomiting. Brain magnetic resonance imaging (MRI) and computer tomography (CT) revealed a space-occupying lesion in the pineal region. The physical examination followed showed that the patient exhibited distinct facial features including a broad and prominent forehead, a long and narrow face, an elongated chin, and sparse frontotemporal hair. Futhermore, the patient presented with intellectual impairment as well as overgrowth indicated by a head circumference of 55.5cm (> 97th centile). At the same time, we pay attention to prenatal ultrasound examination showed widened septum pellucida without any phenotypically relevant findings from chromosome analysis. The patient's family history was unremarkable. On April 28, 2021, the patient underwent neuroendoscopic third ventriculostomy and microscopic pineal lesion resection under general anesthesia (Fig. 1A-F). The sagittal T1/T2-weighted images showed that the tumor was nearly isointense relative to gray matter in the patient (Fig. 1A-B), and sagittal T1-weighted gadolinium-enhanced image showed homogeneous enhancement of the tumor, suggesting a diagnosis of PB (Fig. 1C). Hematoxylin–eosin (HE) staining (Fig. 1G) and immunohistochemistry staining were performed on the pineal mass which showed SYN( +), CGA(-), NSE( +), NF(-), S-100(-), PHOX2B(-), H3K27M(-), VIM(-), INI-1( +), CD99(-), DES(-), Myogenin(-), Vimentin(-), GFAP(-), Nestin(-), Ki67 proliferation index was determined to be 40%, indicating a diagnosis of pinealoblastoma.

NGS was performed on tissue and blood samples using a panel comprising 539 cancer-related genes (Simceredx). Comprehensive genomic profiling of the tumor revealed no SNVs or short insertions/deletions; however, frequent copy number abnormalities were detected including focal gains in chromosomes 3, 7, 8, 9, 13 as well as focal deletions in chromosomes 6, 11, 16 (Fig. 1I). A heterozygous germline NM_022455.4 (*NSD1*): c.5510-1G > A (p.?) mutation was detected in blood samples (Fig. 1H). Further analysis of parents by Sanger sequencing confirmed that the *NSD1* likely pathogenic variant was de novo (Fig. 1J)*.* The parents of the patient refused postoperative chemotherapy, and the infant died of tumor recurrence after 2 months.

The c.5510-1G > A mutation is located in the acceptor splice site in intron 16 of the *NSD1* gene, which is predicted to affect mRNA splicing and result in a significantly altered protein structure. This variant has been recorded as “Likely pathogenic” in Clinvar Database (Variation ID: 2,637,360). Other splice mutations, such as NM_022455.5 (*NSD1*): c.5892 + 1G > A (p.?) and NM_022455.5 (*NSD1*): c.6151 + 1G > A (p.?) are also recorded as pathogenic variants. The donor and acceptor splice site variants typically lead to loss-of-function of the protein [10], with loss-of-function variants in *NSD1* being associated with Sotos syndrome [11]. Furthermore, this specific variant has not been identified in population databases such as gnomAD, indicating its rarity among individuals. Taken together, the mutation was classified as likely pathogenic according to the criteria of the American College of Medical Genetics and Genomics (ACMG).

## Disscusion

Sotos syndrome is characterized by three cardinal features, including characteristic facial dysmorphism, learning disability, and childhood overgrowth in 90% of affected individuals. Additionally, variable minor features were also present [12]. The diagnosis of Sotos syndrome can be confirmed through molecular genetic testing that identifies a heterozygous *NSD1* pathogenic variant or deletion *NSD1* in the proband. This autosomal dominant genetic condition typically presents as a nonfamilial fully penetrant disorder consistent with a de novo mutation in the patient.

Individuals with Sotos syndrome are predisposed to various types of cancer, which generally occurr during childhood. The risk of developing neoplasms in Sotos syndrome is approximately 2–7% [5]. Reported malignancies associated with Sotos syndrome include wilms tumor, vaginal carcinoma, hepatocarcinoma, cavernous hemangioma, neuroectodermal tumor, ganglioglioma, small cell lung carcinoma, neuroblastoma, acute lymphoblastic leukemia, and non-Hodgkin lymphoma [13, 14]. As a rare but aggressive tumor of the pineal gland, although germline mutations in *RB1* and *DICER1* predispose children to PB, but it has not previously been related to *NSD1* [15]. PB displays a pattern of genomic imbalances [16]. Cytogenetic studies have shown structural rearrangements of chromosome 1 and losses involving chromosomes 9, 13, and 16 [17–20]. Tumor molecular groups are enriched for distinct driver gene alterations and broad chromosomal copy-number aberrations [21]. Our patient also exhibited frequent genomic copy number abnormalities, such as focal gains in chromosomes 3, 7 and focal deletions in chromosomes 16. Pineoblastoma was is divided into Pineoblastoma, MYC/FOXR2 activated, Pineoblastoma, RB1-altered, Pineoblastoma miRNA-1 and miRNA-2 base on a desirable diagnostic method of DNA methylation profiling [22]. Unfortunately, our patient has passed away and we do not have available samples for DNA methylation profiling.

Most childhood cancers exhibit significantly fewer somatic mutations compared to adult cancers, but have a higher incidence of germline alterations in cancer susceptibility genes [23]. Certainly, further studies are required to explore the possibility of *NSD1* mutation and their possible contribution to the predisposition to PB. However, knowledge of PB and use of genetic analyses to confirm the specific disorder are important to improve patient care and prevent complications and cancer predisposition. Overall, this is the first report describing PB in a patient who manifested typical clinical features of Sotos syndrome.

## Conclusion

In summary, the identification of a novel de novo* NSD1* likely pathogenic variant in our patient expands both the mutation and phenotype spectrum associated with Sotos Syndrome, thereby providing valuable insights into the potential mechanism underlying of pinealoblastoma pathology.

## Data Availability

The original contributions presented in the study are included in the article. The datasets generated and/or analysed during the current study are available in the ClinVar repository, and may be found at the following address: https://www.ncbi.nlm.nih.gov/clinvar/variation/2637360/. The variant ID is: 2637360. Further inquiries can be directed to the corresponding author.

## Abbreviations

ACMG: American college of medical genetics and genomicse
NSD1: Nuclear receptor SET domain-containing protein 1
NGS: Next genetic sequencing
PB: Pineoblastoma
SYN: Synaptophysin
CGA: Chromogranin-A
NSE: Neuron specific enolas
NF: Neurofilament
S-100: S100 calcium binding protein B
H3K27M: Histone H3-K27M Mutation
VIM: Vimentin
INI-1: Integrase interactor 1
CD99: Cluster of differentiation 99
DES: Desmin
GFAP: Glial fibrillary acidic protein
Ki67: Proliferation cell nuclear antigen, Ki-67
